# Peer-led exercise program for ageing adults to improve physical functions - a randomized trial

**DOI:** 10.1186/s11556-021-00257-x

**Published:** 2021-02-11

**Authors:** D. R. Bouchard, J. V. Olthuis, V. Bouffard-Levasseur, C. Shannon, T. McDonald, M. Sénéchal

**Affiliations:** 1 Cardiometabolic Exercise & Lifestyle Laboratory, Fredericton, NB Canada; 2 Faculty of Kinesiology, University of New Brunswick, Fredericton, NB E3B 4J9 Canada; 3 Department of Psychology, University of New Brunswick, Fredericton, NB Canada; 4 Secteur Éducation et kinésiologie, Université de Moncton, Fredericton, NB Canada; 5 Faculty of Law, University of New Brunswick, Fredericton, NB Canada

**Keywords:** Physical function, Peer-led, Ageing adults, Physical activity, Exercise

## Abstract

**Background:**

A peer-led exercise program is one way to empower people sharing similar characteristics to encourage others to be active, but there is a lack of evidence that these programs have physical function and other benefits when delivered to ageing adults.

**Methods:**

This randomized controlled trial lasting 12 weeks proposed an exercise peer-led program offered to 31 adults aged 50 and above, twice a week, by a trained leader of the same age from March to May 2019. The program was offered for free with limited space and equipment. Valid tests of physical function (e.g., 30-s chair stand, 6-min walk test) were used to assess the functional benefits. Psychosocial outcomes were assessed using self-reported questionnaires and metabolic outcomes via a fasted blood draw.

**Results:**

A significant difference was found between pre-and post-values in most physical function tests in the intervention group (all *p* < 0.05). When adjusted for potential confounders, the intervention group was significantly associated with a more significant improvement on the chair stand test (ß = .26; *p* < 0.001; *r*^*2*^ = 0.26), the arm curl (ß = .29; *p* < 0.001; *r*^*2*^ = 0.49), as well as the 6-min walk test (ß = -.14; *p* < 0.001; *r*^*2*^ = 0.62) compared with the control group. Using repetitive measures generalized linear model, the interaction between the changes and the group was significant for all three tests. Benefits were also observed for participants’ stress level and perceived health in the intervention group compared to the control. Finally, no significant difference was observed between groups for metabolic health.

**Conclusions:**

The current work suggests that a 12-week peer-led exercise program can improve physical function for adults age 50 and above.

**Trial registration:**

NCT03799952**(**ClinicalTrials.gov) 12/20/2018.

**Supplementary Information:**

The online version contains supplementary material available at 10.1186/s11556-021-00257-x.

## Background

The number of ageing adults is increasing worldwide [1]. Even though there are many known physical and mental health benefits to regular physical activity [2], most adults do not adhere to an active lifestyle. For example, less than 16% of Canadian adults aged 18–79 reach the national physical activity guidelines when measured objectively [3]. Some have suggested that receiving the exercise program through a peer-leader could lead to benefits by increasing adherence [4].

A peer-leader is a person who shares similar experiences and status to those they are leading [5]. Peer-led exercise programs have shown success in encouraging physical activity [6–8]. This model of delivery is affordable [8], gives a sense of empowerment [9], and promotes social connectedness [7]. One study even concluded that peer-led exercise programs were as effective as professionally-led exercise programs in the community [6]. However, a systematic review in 2018 reported that although peer-led exercise can promote and maintain adherence to exercise programs, the peer-led exercise programs’ impact on physical function was still unclear [10]. Quantifying the impact of peer-led exercise on physical function is essential. It has direct implications for policy and practice decisions about promoting participation in these programs among ageing adults. It is also crucial for funding to support or to subsidize peer-led programs.

### Objectives

This study’s main objective was to test the change in participants’ physical function, measured by the changes on the 30-s chair stand test, in a 12-week, peer-led exercise program offered to community adults (age 50 and above) as compared to a control condition. The secondary objective was to explore the other physical function tests, and the peer-led exercise program’s psychosocial and metabolic benefits.

## Methods

### Trial design

Participants were randomized to receive either the intervention (Spring) or waitlist control (Fall) using concealed envelopes prepared by a third party using a closed container. That container included all 62 papers. A student not related to the project performed the allocation. Peer leaders were blinded to randomization as they did not know that participants in the Spring groups were in the intervention group, and participants in the Fall were in the control group. Participants in the Spring were also invited to participate again in the Fall. Peer-leaders were told that we were limiting the number of participants for space and to start with smaller group sizes for the Spring groups. The participants were blinded as they were not tested after the Fall session, but it was not mentioned.

### Recruitment

To be eligible to participate, individuals had to be at least 50 years, could physically come to the location where the exercise sessions were delivered, be cleared to exercise as determined by the Canadian Society of Exercise Physiology (CSEP) Get Active Questionnaire [11] or by a physician if needed, and participants needed to consider themselves as inactive by confirming not currently being involved in organized physical activity program. Participants were excluded if they were not cleared by a physical activity screening test or did not receive clearance from their primary physician to participate. Finally, participants were not eligible if they participated in the offered peer-led exercise program in the past. The tested peer-led exercise program has existed for many years but has not been appropriately evaluated. Therefore, the same inclusion/exclusion was used, which includes the threshold for age 50+. Initially, the program was developed to promote independence and prevent falls by long-term participation. Participants were recruited through radio advertisements, newspapers, posters, and social media.

Four peer leaders were recruited using the same strategy based on a first-come, first-served procedure. They were eligible if they reported being a regular exerciser, willing to participate in 32 h of interactive training sessions offered by the provincial fitness accreditation body over 4 days, and willing to volunteer to offer an exercise program in their community for free. Peer leaders received a manual, along with practical training. A formal background in health or fitness was not required. Continued support was offered to the leaders once they began leading the program. This support was offered through both the provincial fitness accreditation body and the research staff.

Recruitment for all participants (intervention and control) occurred between January and March 2019. When participants called to enrol in the program, they were assessed for eligibility over the phone by the research staff. Those who were eligible were told that they would be randomized into either the Spring (intervention) or the Fall (control) group, but testing would only occur in March and May 2019. The exercise program was offered between March and May 2019 for those randomized to the intervention. It was offered between September and December 2019 for those randomized to the control condition.

### Intervention

The exercise program, called Zoomers on the Go [12], occurred at an indoor community location (e.g., community room, church basement) at no cost to participants. The peer-led exercise program was offered twice a week for 60-min (10-min warm-up, 10-min aerobic exercise, 10-min balance exercises, 15-min of muscle strengthening exercises, 10-min of flexibility activities, and a 5-min cool-down) for 12 weeks. Resistance exercises were done using a coloured TheraBand, a 9-in. sponge ball, and paper plates. There were also chairs available for every participant if needed. More details on the program are presented in the supplementary file.

### Primary outcomes

The primary outcome was strength assessed by the change on the 30-s chair stand test. Participants were asked to sit on the edge of a chair, standing up and sitting down as many times as possible in 30 s. The research assistant recorded the number of repetitions completed during the test [13].

### Exploratory outcomes

Besides the 30-s chair stand test, three other Senior Fitness Tests (SFT) were used to explore the program’s potential benefits on physical function. These were the 6-min walk test (6MWT), the 30-s arm curl test and the Back Scratch Test. In addition to the SFT battery of tests, the one-leg stance was used to assess a participant’s balance, eyes open, and eyes closed [14]. Finally, grip strength was collected using a JAMAR analogue handheld dynamometer (Lafayette Instrument Company, USA) for both left and right hands. The highest value of each hand was added together, according to CSEP [14].

Capillary blood sampling was conducted using the CardioCheck Analyzer device to determine high-density lipoprotein (HDL), triglycerides, low-density lipoproteins (LDL), and glucose [15].

Psychosocial outcomes were assessed via questionnaire. The Depression Anxiety Stress Scales - 21 item (DASS-21) was used to measure past week depression (scores of 13, 20, 27, and 38 indicate mild, moderate, severe, and extremely severe symptoms, respectively), anxiety (scores of 8, 10, 15, and 20 indicate mild, moderate, severe, and extremely severe symptoms, respectively), and stress (scores of 15, 19, 26, and 34 indicate mild, moderate, severe, and extremely severe symptoms, respectively) [16]. The Short Form Health Survey - 36 items (SF-36) was used to measure day-to-day functioning and quality of life [17]. The scale is composed of eight domain subscales (i.e., Physical Functioning, Role Limitations due to Physical Health, Pain, General Health, Energy/Fatigue, Social Functioning, Role Limitations due to Emotional Problems, Emotional Well-Being) scored from 0 (worst) to 100 (excellent).

### Potential confounder outcomes

Demographic data, including age, sex, marital status, occupation, and household income, were assessed via a self-report questionnaire.

Attendance at the peer-led exercise group was collected on-site by the program leader. The maximum number of sessions was 23 as one session was cancelled for a holiday.

Body weight was measured to the nearest 0.1 kg, and height was measured to the nearest 0.1 cm on a calibrated column scale (SECA model #213, Hamburg, Germany), using CSEP protocols. Body-mass index (BMI) was calculated using body weight, height and the CSEP equation for BMI. Resting blood pressure and resting heart rate were collected on-site with a portable blood pressure cuff (Omron M1 Plus -HEM-4011C-E).

Physical activity level was objectively assessed using PiezoRxD pedometers (Steps Count, CA) to describe the sample at baseline. Participants were asked to wear the pedometer for seven consecutive days before the start of the program. The pedometer was used to track steps per day and estimate total time spent participating in moderate to vigorous physical activity based on walking cadence. Total time spent at a cadence of a minimum of 120 steps per minute was considered time spent in moderate and vigorous-intensity, respectively [18].

### Sample size calculation

The effect size expected on the chair stand test was determined using clinical data previously collected on 248 participants participating in this program. Despite the high number of participants, this dataset lacked a control group, and testing was done in a clinical setting without rigid testing sessions to respect the 12-week intervention. Some participants who saw changes in the chair stand participated in the program many years before being tested. Nonetheless, an improvement of 1.72 s ± 2.10 s was observed for the sit-to-stand test (standing from a seated position as fast as possible for five repetitions). Assuming the same proportion as an effect size, that the control group would not improve, with a power of 80% and an alpha of 95%, it was estimated that 24 people per group were required to observe the same proportion of improvement on the sit-to-stand test. To account for the anticipated drop-out rate (i.e., 30%) [19], 31 participants were recruited per group.

### Data analytic plan

Differences between groups on descriptive variables and outcomes at baseline were tested via T-tests and Chi-square tests depending on the variable’s nature. Changes in physical function were tested using linear regression models. This was done using a stepwise strategy, with the dependent variable being the change pre-post. The independent variables were the treatment group, baseline value on the test, and any differences between groups observed at baseline on descriptive characteristics. General Linear Models repetitive measure tests were used to test how pre and post observations on each functional test were affected by the interaction group*time adjusted for baseline differences in descriptive characteristics. We also explored if the number of sessions attended by participants in the intervention group predicted changes in physical function using linear regression models once adjusted for potential confounders.

## Results

**Fig. 1:**
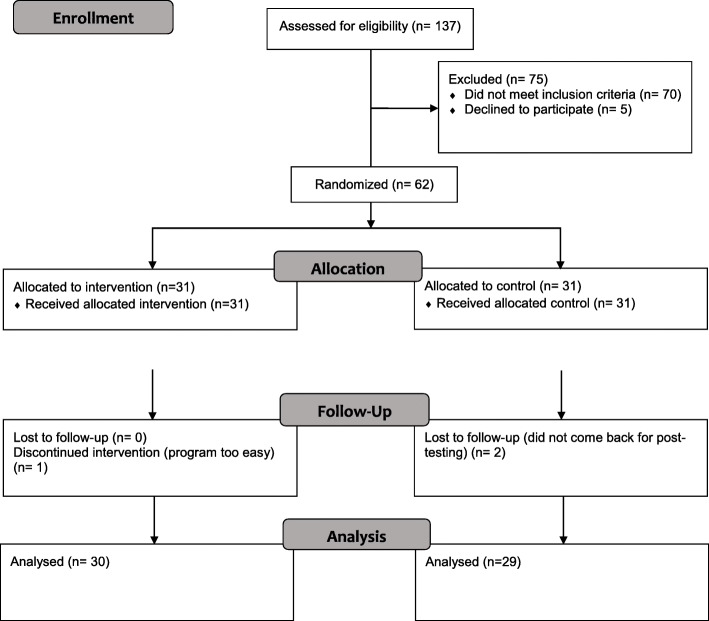
CONSORT flow diagram of enrollment

Sixty-two participants took part in the study (31 intervention, 31 control). Three participants (4.8%; one intervention and two control) dropped out, leaving 59 for analysis (see Fig. 1). Participants reported dropped out because the program was too easy; they had an additional family commitment, or did not want to attend the post-testing session.

Participants were an average age of 66.1 and 66.6 in the control and intervention groups, respectively. Of those participants, women made up 79.3% in the control group and 93.3% in the intervention group. Descriptive information of the sample for participants who completed the intervention is presented in Table 1. At baseline, no difference besides BMI was observed between the two groups on descriptive variables.

**Table 1:** Descriptive characteristics of participants

	Control (*n* = 29)	Intervention (*n* = 30)	*p* value
**Age (years)**	66.1 ± 7.0	66.6 ± 7.2	0.79
**BMI (kg/m**^**2**^**)**	25.8 ± 4.8	28.9 ± 4.5	0.02
**Women**	23 (79.3)	28 (93.3)	0.12
**Occupation (Retired)**	14 (48.3)	19 (63.3)	0.15
**Marital Status (Married)**	20 (69.0)	14 (46.7)	0.24
**Household Income (>$100,000/ year)**	6.0 (21.0)	6.0 (20.0)	0.79
**Physical Activity Level (Steps/day)**	7221 ± 2964	6349 ± 2903	0.27
**Physical Activity Level (MVPA/week)**	48 ± 81	68 ± 68	0.51

Data are presented as average ± SD or N (%)

Chi Square tests were used to test for potential differences among groups for categorical variables while T-test were used to test for potential differences for continuous variables among groups

Attendance to the exercise sessions averaged 15 ± 6 sessions out of 23 sessions (68%) for participants in the intervention group who completed the post-testing. The number of sessions attended was not associated with any physical function changes when adjusted for age, sex, and baseline value of each physical function.

There was a significant within-group difference found between pre-and post-values in all physical function tests (*p* < 0.05), except for balance (eyes closed) and back scratch test for the intervention group (Table 2). There was group effect for the chair stand (ß = .26; *p* < 0.001; *r*^*2*^ = 0.26), arm curl (ß = .29; *p* < 0.001; *r*^*2*^ = 0.49), and 6-min walk test (ß = .14; *p* < 0.001; *r*^*2*^ = 0.62) when models adjusted for baseline value, sex, age and BMI. When using GLM repetitive measures, the interaction group *time for the chair stand (*F* = 13.85), the arm curl (*F* = 14.06) and the 6-min walk (*F* = 16.79) was significant (all *p* < 0.001).

**Table 2:** Functional Outcomes

	Control (*n* = 29)	Intervention (*n* = 30)		
	Pre	Post	Pre	Post
Chair stand (reps/30s)	13.6 ± 2.7	14.1 ± 3.5	14.1 ± 2.6	17.9 ± 4.4*Γ
Arm curl test (reps/30s)	17.3 ± 4.1	17.4 ± 3.5	20.1 ± 5.1**Γ**	23.9 ± 4.9*Γ
Handgrip strength (kg)	51.4 ± 15.5	50.1 ± 17.6	51.8 ± 14.9	55.3 ± 15.4*
Back scratch test (cm)	− 5.2 ± 9.7	−4.7 ± 9.9	−7.5 ± 9.9	− 5.7 ± 9.9
Timed up and go (s)	5.3 ± 0.99	4.9 ± 1.1*	5.3 ± 1.2	4.7 ± 0.9 *
6-min walk test (m)	507 ± 90.3	504 ± 79.7	476 ± 94.3	539 ± 82.5*Γ
Balance (eyes opened) (s)	27.9 ± 16.4	29.7 ± 15.6	26.7 ± 14.4	34.7 ± 13.4*Γ
Balance (eyes closed) (s)	4.9 ± 3.2	4.4 ± 2.9	5.0 ± 3.4	5.3 ± 3.5

Data are presented as average ± SD *significant pre-post changes within groups (*p* < 0.05)

Γ significant difference in change between intervention and control groups (*p* < 0.05)

For psychosocial outcomes (Table 3), there was a significant within-group difference found between pre-and post-intervention past week stress symptoms (*p* < 0.05) for those in the intervention. There was also a group effect for the past week’s stress symptoms (*p* = 0.01). On the SF-36, there was a group effect on General Health, Energy/Fatigue, and Role Limitations due to Emotional Problems, all of which improved significantly more in the intervention than control groups (all *p* < 0.01). Finally, in terms of metabolic outcomes (Table 4), no differences in pre-post changes were observed between the two groups. However, some improvements were observed for both groups within-groups (e.g., on resting HR, diastolic BP, and glucose).

**Table 3:** Psychosocial outcomes

	Control (*n* = 29)		Intervention (*n* = 30)	
	Pre	Post	Pre	Post
The Depression Anxiety Stress Scales				
Stress (0–14 ‘normal’)	5.7 ± 6.2	8.2 ± 7.7	6.0 ± 5.6	4.1 ± 3.9*Γ
Anxiety (0–7 ‘normal’)	2.6 ± 4.7	5.0 ± 7.5	3.5 ± 3.8	3.2 ± 3.3
Depression (0–9 ‘normal’)	3.2 ± 3.8	5.5 ± 9.1	4.8 ± 6.3	3.2 ± 5.6
Short Form Health Survey (0–100; 100 being best)				
Physical Functioning	80.9 ± 20.1	80.4 ± 18.8	78.0 ± 21.2	81.2 ± 20.2
Limitations due to Physical Health	81.7 ± 28.8	79.0 ± 32.8	78.3 ± 33.9	90.8 ± 24.1*
Pain	79.1 ± 17.6	71.0 ± 23.0*	73.5 ± 22.3	71.9 ± 20.8
General Health	72.4 ± 11.8	69.8 ± 16.4	71.2 ± 19.1	75.5 ± 15.6*Γ
Energy/Fatigue	67.4 ± 18.0	65.4 ± 17.6	63.5 ± 19.1	68.7 ± 16.3*Γ
Social Functioning	88.9 ± 16.3	86.4 ± 19.1	90.2 ± 14.9	92.1 ± 13.7
Limitations Emotional Problems	78.8 ± 33.8	84.0 ± 32.1	84.6 ± 28.3	88.9 ± 25.3*Γ
Emotional Well-Being	82.5 ± 12.4	78.7 ± 13.2	80.4 ± 13.5	84.7 ± 10.2

Data are presented as average ± SD

*significant pre-post changes within groups (*p* < 0.05)

Γ significant difference in pre-post change between intervention and control groups (*p* < 0.05)

**Table 4:** Metabolic Outcomes

	Control (*n* = 29)		Intervention (*n* = 30)	
	Pre	Post	Pre	Post
Resting HR (beats/min)	68.1 ± 9.4	63.0 ± 7.0*	70.5 ± 6.7	67.2 ± 7.6*
Systolic blood pressure (mmHg)	123.8 ± 12.6	125.7 ± 14.9	127.5 ± 11.1	124.5 ± 9.5
Diastolic blood pressure (mmHg)	77.0 ± 7.8	72.0 ± 8.9*	77.0 ± 6.8	74.5 ± 6.3*
HDL cholesterol (mmol/L)	1.83 ± 0.41	1.74 ± 0.37*	1.47 ± 0.86	1.45 ± 0.27
Triglycerides (mmol/L)	1.30 ± 0.64	1.24 ± 0.48	1.91 ± 1.12	1.66 ± 0.68
LDL cholesterol (mmol/L)	2.88 ± 1.0	3.2 ± 0.77	2.83 ± 1.08	3.03 ± 0.94
Glucose (mmol/L)	5.34 ± 0.69	4.70 ± 0.52*	5.61 ± 1.05	5.11 ± 0.65*

Data are presented as average ± SD

*significant pre-post changes within groups (*p* < 0.05)

## Discussion

The current study results support the idea that peer-led exercise programs for aeging adults can lead to physical function improvements. Like previous studies [10, 20, 21], the drop-out rate for this program was meagre with a relatively high attendance rate. The combination of low drop-out rates and significant physical function improvements suggests that peer-led exercise programs are a successful intervention for ageing adults living in the community.

Other studies have reported diverse findings [10] on peer-led exercise program’s ability to improve physical function. Although the effects of peer-led exercise programs have been systematically reviewed, it is essential to note that many program and participant characteristics can lead to different physical functions. Compared with previous studies looking at the improvement in physical function following a peer-led exercise program, our study involved younger participants (average 66 years) with relatively good health compared to the studies reported in Burton et al. (2018) [10]. Participants in the present study could exercise at a greater intensity than studies involving older participants who observed lower physical function improvements. In support, Dogro et al. (2009; average age 69 years old) reported more improvement for participants who started the program with a greater fitness level [22]. Another essential feature of a peer-led exercise program that leads to physical function improvement seems to be formal training for leaders [8, 22, 23]. It is possible that serious training for peer-leaders, as in the present study, is needed to draw significant physical function benefits.

When discussing peer-led exercise programs, it is essential to note that the literature reports the effects of both peers who deliver the exercise program and peers who encourage ageing adults to become more active. Previous studies have reported that delivering the exercise program by peers is more effective than peers motivating other ageing adults to become more active [24, 25]. It is thus essential to differentiate the two strategies when combining the benefits of a peer-led strategy.

Participants in the intervention group increased the chance of improving their performance on the chair stand, the 6-min walk test, and the arm curls tests by 14 to 29% regardless of their age, sex, and baseline performance on the test or initial physical activity level. However, the variability at baseline was pretty small, suggesting that participants were already reasonably fit before the intervention. When looking at all participants’ percentile level for each test based on age group and sex [13], the average was 55%. This observation suggests that this peer-led exercise program could be more useful for people with lower physical function at baseline, reported before [26]. It is possible that ageing adults who are already more physically active than the average are those who seek out these community programs. Nonetheless, an increase in physical function could lead to greater life expectancy and living independently for a more extended period, as suggested in the literature [27].

The minimally clinically important difference (MCID), within a clinical setting, is the smallest benefit of value to the patient and is not just based on statistical significance but meaningful changes to the individual [28, 29]. MCID values vary depending on the physical function test and the characteristics of participants. Interestingly, the MCID values for people without clinical conditions are hard to find in the literature. One study conducted with frail ageing adults suggests an MCID of an improvement in 17.8 m in the 6-min walk test would be clinically significant [30]. While the sample varies from the current study, our findings suggest a mean improvement of 63 m, well above the MCID for the intervention group. Similarly, another study involving ageing adults with COPD [31] reported that the MCID for the 30-s sit-to-stand test would be two repetitions. In comparison, another research conducted with adults undergoing vestibular rehabilitation suggests an improvement of 2.3 s is clinically meaningful [32], and the intervention group improved the number of stands by 3.8 repetitions. This suggests statistically significant improvement and the clinical significance for ageing adults who participated in this peer-led exercise program.

Findings suggest no significant intervention-specific improvement for metabolic health outcomes, but other studies had reported similar findings when baseline values were relatively average, as in the present study [33]. In contrast, although the present sample reported ‘normal’ psychosocial functioning at baseline, a more significant improvement was observed in stress for participants in the intervention vs control. Participants in the intervention also reported a more significant improvement in general health, more energy, and fewer role limitations due to emotional problems than those in the control group. A study conducted with 78 ageing adults who participated in a peer-led exercise program also reported an improvement in many domains of the SF-36 despite high baseline scores [21]. According to previous studies, a greater sense of self-efficacy arising from peer-led exercise could, in part, explain a change in health perceptions [9]. Like the metabolic outcomes, a lack of change in anxiety and depressive symptoms in the present study is likely due to the participants’ non-clinical nature at baseline. It is possible that ageing adults with greater psychosocial difficulties at baseline would have experienced more significant improvements; however, it is also likely that such individuals would find it more challenging to initiate participation in a peer-led exercise group.

### Implications

Peer-led exercise programs can empower seniors to serve their community. This model of exercise delivery is feasible and can be offered at a low cost. In Zoomers on the Go, the only cost was the provincial fitness accreditation training for leaders and the equipment ($15 per participant). The registration was done by non-profit organizations who also identified a room to offer the free exercise program. The next steps of this program are to impliment the model to be sustainable and study if it is possible to offer it remotely to reach more individuals.

### Limitations

The majority of participants were women suggesting that interventions should be implemented to attract more men to peer-led exercise programs, perhaps by offering an exclusive class for men. The fact that people needed to come to the university facilities for testing two times and regularly attend two weekly exercise sessions may have limited the accessibility of the program for people with lower socioeconomic status. Emotional health was in favour of the control group, compared to the intervention.. Despite these limitations, the study design and the possibility of large-scale implementation of the program are strengths that counterbalance the weaknesses.

## Conclusion

The current work demonstrates the efficacy of a peer-led exercise program in improving physical function health among ageing adults. This finding is relevant because of the strong association between physical function and important outcomes such as fall rate, institutionalization, or premature mortality for ageing adults. Future studies need to evaluate the cost-effectiveness of peer-led exercise programs, look at strategies to offer these programs in remote areas, and identify how to attract more men.

## Supplementary Information

**Additional file 1.**

## Data Availability

The datasets used and analyzed during the current study are available from the corresponding author on reasonable request.

## Abbreviations

RCT: Randomized controlled trial
SFT: Senior fitness tests
GLM: Generalized linear model
CSEP: Canadian society of exercise physiology
6MWT: 6-Min walk test
HDL: High-density lipoprotein
LDL: Low-density lipoproteins
DASS-21: Depression anxiety stress scales - 21 item
SF-36: The short form health survey - 36 items
MCID: Minimally clinically important difference
