# Supplementary material for: Peer-led exercise program for ageing adults to improve physical functions - a randomized trial
Source: Eur Rev Aging Phys Act. 2021 Feb 11;18:2. doi: 10.1186/s11556-021-00257-x (PMC7879524; doi:10.1186/s11556-021-00257-x)
Supplement: Supplementary file 1 — Additional file 1. [file 11556_2021_257_MOESM1_ESM.docx]

**Zoomers on the Go – Supplementary Document**

| **Warm-up** | |
| --- | --- |
| Warm-up includes organized movement of lower and upper  extremities with good alignment and a stable core. Include all  planes (front/side/back of body) of movement to lubricate joints. | |
| Arm actions: can be used for assistance (wall or chair), can mirror legs or be opposite to the legs. | Experiment with a variety of arm movements:   - Ski arms - Washing windows - Kayak arms - arm circles backwards (small to big) - swimming strokes - crossing arms in the front - moving them to the side, up and down (but below the heart in warm-up) - Bend and straighten the elbows, wrist circles, each finger to thumb.   Emphasis on opening the chest and squeezing the shoulder blades. |
| Leg Warm-Up Movements while walking or on the spot | Experiment with a variety of arm movements:   - Alternate heel and/or toe touch front - Alternate heel and/or touch front diagonal - Alternate foot tap laterally (side) (more advance people can perform alternate side leg lift, lifting the leg directly to the side, foot off the floor) - Alternate posterior (behind) leg push (hip extension) with emphasis on gluteal squeeze to strengthen gluteal and hamstrings. Knee of the supporting leg is unlocked and strong. - Alternate hamstring curls. |

| **Aerobic** | |
| --- | --- |
| **Notes for Leaders** | **Example** |
| Cardiovascular can be identical to the warm-up with more intensity (10 Min).  Near the end of the cardio segment, lower the intensity to help prepare for the next class  segment. | |
| Cardiovascular segment can be very similar to the warm-up; add more intensity (walking  faster, bigger Range of Motion (ROM) during this segment. To increase intensity, the legs have to move more! | Various leg movements and patterns can include (similar to warm-up, with more  intensity): alternate heel touch front, alternate heel touch front, alternate toe taps laterally, alternate posterior leg push (hip extension, toes touching the floor).   - Narrow step walk - Wide step walking - Combined narrow and wide step walking - Increase or decrease number of steps per gait pattern - Step to walking: Moving forward, take a long step with one leg and bring the other leg up beside the first leg. Repeat with other leg. Focus on hip extension, emphasize gluteal squeeze. - Forward heel walking - Forward toe walking - Combination of toe and heel walking - Walk sideways, using a sidestep (not in a circle). (EX: 2 side touches, 2 side squats) - Walking forward, add an alternate leg curl movement with each leg.   Verbal cues: Walk tall, head erect, ears over shoulders. Eyes focus straight ahead; focus  on a target in front of you. Abdominal muscles engaged, chest slightly lifted, shoulder blades back and down.  Cardiovascular cool-down: slow walking around the room while gently swinging the  arms. After a few minutes, marching on the spot taking a few deep breaths. |

| **Strength** |
| --- |
| **Notes for Leaders** |
| Chest  1 set of 15 repetitions  Chest Press with Exercise Band |
| Upper Back/Shoulder  1 set of 15 repetitions  Seat Row with Exercise Band |
| Buttocks Front of Thighs (quads) Back of thighs (hamstrings)  1 set of 15 repetitions  Squats |
| Core Work  1 set of 15 repetitions  Modified Superman with chair |
| Calves  1 set of 15 repetitions  Heel raises |
| Shins (front of lower leg)  1 set of 15 repetitions  Toe Raises |
| Outer Thigh  1 set of 15 repetitions  Standing ball abduction against the wall |
| Inner Thigh  1 set of 15 repetitions  Ball Squeeze Seated |
| Biceps (if time permits)  1 set of 15 repetitions  Bicep curls with band seated |
| Triceps (if time permits)  1 set of 15 repetitions  Triceps extension with the band seated |

| **Flexibility/Balance** | |
| --- | --- |
| **Notes for Leaders** | **Example** |
| 1) Free leg | Stand and hold. Near wall or chair. Free standing, hand not touching the chair unless necessary. Bend non supporting leg at the knee (comfortable near 90 degrees), kneecap pointing to the floor. Rotate ankle of bent leg. Stand 15 to 30 seconds. Change side. |
| 2) Free leg: | Touch front, center, back, center (repeatedly, slowly and with control). Use chair for support if necessary. Change sides after 15 to 30 seconds.  PROGRESSION BELOW:   - Same exercise as above, don’t hold on to the chair (if possible). - Same exercise as above, hold on to the chair, but foot does not touch the floor. - Same exercise as above, don’t hold on to the chair, foot does not touch the floor. |
| 3) Tandem walking | Walking with one foot in front of the other like walking on a tight rope or balance beam. Use chair or wall for support if needed. |
| 4) Bicycling movement with one leg | Use chair or wall for support if needed. (if time permits) |

| **Cool down** |
| --- |
| Stretches (5 to 10 minutes) from pages 195 to 202.  Please note that on some pages, the stretches are for the same muscle group; just  with some modifications. For example, just do ONE of the stretches,  not all three. Always start with the first option if possible.  Stretching should be completed at the end of every class.  Finish with stretches on the chair. Review in your manual. |
| **Notes for Leaders** |
| Front of thighs & hip flexors |
| Back of the thigh and calves |
| Inner thigh stretch |
| Outer thigh stretch |
| Chest stretch |
| Upper back stretch |
| Side of the body stretch |
| Front of the upper arm and front shoulder stretch |
| Back of the upper arm and back shoulder stretch |
| Back of the upper stretch |
| Back of neck stretch |
| Side of neck stretch |

| **Equipment** |
| --- |
| Resistance Band |
| 9 inch Exercise Ball |
| Paper Plates |
